# Supplementary material for: Dynalign II: common secondary structure prediction for RNA homologs with domain insertions
Source: Nucleic Acids Res. 2014 Nov 21;42(22):13939–48. doi: 10.1093/nar/gku1172 (PMC4267632; doi:10.1093/nar/gku1172)
Supplement: SUPPLEMENTARY DATA [file supp_gku1172_nar-02021-z-2014-File012.zip › manual/Text/stochastic.html]

RNAstructure Command Line Help: stochastic


|  |  |  |
| --- | --- | --- |
|  | RNAstructure Command Line Help  stochastic and stochastic-smp | - Contents - Index |
| stochastic is used to sample secondary structures from the Boltzman enemble. The probability of sampling a given structure is proportional to the probability that it occurs. stochastic-smp is a parallel processing version for use on multi-core computers, built using OpenMP. USAGE: stochastic <input file> <ct file> [options] OR: stochastic-smp <input file> <ct file> [options] Required parameters: <input file> \| The name of a file containing input data. This input data can be in one of two formats: 1. Partition function save file (holds base pairing probability data for all pairs and can be generated using the partition interface). 2. Sequence file (holds raw sequence: .seq or .fasta). Note that lowercase nucleotides are forced single-stranded in structure prediction. Note that in order to use a squence file, the "sequence" flag must be specified (see "--sequence" below).; <ct file> \| The name of a CT file to which output will be written. Options which don't require added values: -d, -D, --DNA \| This flag only matters if the input file is a sequence file and has been identified as such (see "--sequence" below). Specify that the sequence is DNA, and DNA parameters are to be used. Default is to use RNA parameters.; -h, -H, --help \| Display the usage details message.; --sequence \| Identify the input file format as a sequence file. Options which require added values: -e, -E, --ensemble \| Specify the ensemble sample size. Default is 1000 structures.; -s, -S, --seed \| Specify the random number seed as an integer. Using a different seed will result in a different sample of structures. Default is 1234. Notes for smp: stochastic-smp, by default, will use all available compute cores for processing. The number of cores used can be controlled by setting the OMP\_NUM\_THREADS environment variable. References: 1. Reuter, J.S. and Mathews, D.H. "RNAstructure: software for RNA secondary structure prediction and analysis." *BMC Bioinformatics*, 11:129. (2010). 2. Harmanci, A.O., Sharma, G. and Mathews, D.H. "Stochastic sampling of the RNA structural alignment space." *Nucleic Acids Res.*, 37:4063-4075. (2009). 3. Ding, Y. and Lawrence, C.E. "A statistical sampling algorithm for RNA secondary structure prediction." *Nucleic Acids Res.*, 31:7280-7301. (2003). | | |
| Visit The Mathews Lab RNAstructure Page for updates and latest information. | | |
